# Assessment of whole genome amplification-induced bias through high-throughput, massively parallel whole genome sequencing

**DOI:** 10.1186/1471-2164-7-216

**Published:** 2006-08-23

**Authors:** Robert Pinard, Alex de Winter, Gary J Sarkis, Mark B Gerstein, Karrie R Tartaro, Ramona N Plant, Michael Egholm, Jonathan M Rothberg, John H Leamon

**Affiliations:** 1 454 Life Sciences, 20 Commercial Street, Branford CT 06405, USA; 2 MB&B Department, Yale University, 266 Whitney Ave., New Haven CT 06520, USA

## Abstract

**Background:**

Whole genome amplification is an increasingly common technique through which minute amounts of DNA can be multiplied to generate quantities suitable for genetic testing and analysis. Questions of amplification-induced error and template bias generated by these methods have previously been addressed through either small scale (SNPs) or large scale (CGH array, FISH) methodologies. Here we utilized whole genome sequencing to assess amplification-induced bias in both coding and non-coding regions of two bacterial genomes. *Halobacterium *species NRC-1 DNA and *Campylobacter jejuni *were amplified by several common, commercially available protocols: multiple displacement amplification, primer extension pre-amplification and degenerate oligonucleotide primed PCR. The amplification-induced bias of each method was assessed by sequencing both genomes in their entirety using the 454 Sequencing System technology and comparing the results with those obtained from unamplified controls.

**Results:**

All amplification methodologies induced statistically significant bias relative to the unamplified control. For the *Halobacterium *species NRC-1 genome, assessed at 100 base resolution, the D-statistics from GenomiPhi-amplified material were 119 times greater than those from unamplified material, 164.0 times greater for Repli-G, 165.0 times greater for PEP-PCR and 252.0 times greater than the unamplified controls for DOP-PCR. For *Campylobacter jejuni*, also analyzed at 100 base resolution, the D-statistics from GenomiPhi-amplified material were 15 times greater than those from unamplified material, 19.8 times greater for Repli-G, 61.8 times greater for PEP-PCR and 220.5 times greater than the unamplified controls for DOP-PCR.

**Conclusion:**

Of the amplification methodologies examined in this paper, the multiple displacement amplification products generated the least bias, and produced significantly higher yields of amplified DNA.

## Background

Continued improvement in sequencing quality, combined with increasingly sophisticated bioinformatic analysis of sequence data, has increased the relevance of whole genome sequencing to many fields of biological science including the pharmaceutical industry, agriculture, national defence and medicine [1]. Similarly, the increased availability of sequence data has served to support increasingly complex and informative comparative genomic studies. In both cases, the enhanced relevance and power of genomic comparisons have, in turn, furthered demand for still more sequence data, with the goal of comparing entire genomes. Although high-throughput sequencing methodologies have been developed to accommodate the demand for sequence output, they consume large amounts of a valuable input: genomic DNA. For example, a Taq-man based whole genome association study of 300,000 SNPs would require approximately 9 mg of genomic DNA, more than obtained in routine clinical blood samples [2]. Less input DNA is required for a genome-wide, microarray-based survey restricted to known mutations, but even this would require some form of amplification step [3].

Other applications also place a high demand on potentially scarce DNA. Emerging relationships between specific genotypes and risk factors or disease states have focused attention on DNA samples that are of great medical/scientific importance, but of limited supply, such as tumor samples, lavages, buccal swabs, or samples generated by laser capture microscopy [4]. While laser capture offers single cell accuracy and both lavages and swabs permit minimal patient invasion and discomfort, these methodologies produce far less genomic DNA than less precise, more invasive techniques [5-7]. Some inherently rare samples, such as difficult to culture micro-organisms [8] or genes from an individual bacterium [9] are of great scientific interest, but cannot be sequenced by current technologies without pre-amplification [10,11]. Considerable interest also exists in sequencing low abundance DNA from museum or fossil specimens, although amplification of these samples must address issues of degradation [12] and contamination [13,14]. High rates of consumption, combined with high demand from the scientific community, may result in hard decisions restricting access to these limited or irreplaceable samples.

Whole genome amplification (WGA) can potentially eliminate DNA as a limiting factor for genetic assays. However, in order to fulfil this role, WGA must satisfy some basic requirements. First, the amplification process should be highly accurate, so as to avoid introducing an undue number of errors. Second, amplification should not induce a bias in the distribution of the product DNA. Third, a high amplification factor is required, so that the WGA generates a useful amount of DNA from small starting samples. Finally, the WGA method should be applicable to a wide array of genomes. For maximal efficiency, the WGA protocol would be universally applicable, without need for separate optimization for each sample. In this paper we will address the latter three points – bias, yield, and applicability to two different genomes – leaving the more complicated studies of both amplification fidelity and the sequence-specific causes of bias for another time.

Three primary forms of WGA have been developed: multiple displacement amplification (MDA) [15,16], primer extension preamplification (PEP) [17], and degenerate oligonucleotide primed PCR (DOP) [18]. These WGA methods have been compared in previous papers, but these comparisons have been limited in scale. The authors either scanned individual nucleotide mutations for SNP analysis, or used comparative genomic hybridization (CGH) or fluorescence in situ hybridization (FISH) to scan large regions of the genome [15,19-22]. In the SNP analyses, the comparison is at a high resolution, but small in scope, while for CGH and FISH, the comparison is at a low resolution (since these methods are extremely forgiving of point mutation errors), but large in scope. The different methodology resolutions can therefore report differing levels of bias, as described in a recent study of ϕ29 fidelity using both direct sequencing and array hybridization of 10000 SNPs [23]. While array-hybridization results revealed whole genome amplification-related loss of 6 regions (approximately 5.56 Mb) and under-representation of another 8 regions, SNP calls from amplified DNA were not statistically different from those of unamplified material [23]. Ideally, any comparison of WGA methods would investigate amplification bias across entire genomes at the highest resolution possible.

For this paper, we have used MDA, PEP and DOP to amplify two bacterial genomes, *Halobacterium *species NRC-1 with a relatively high 66% GC content (derived from the 68% GC main chromosome and two, lower GC content associated minichromosomes, of which at least one, pNRC100, is multicopy [24] – see next section for more detail) and *Campylobacter jejuni *with a single, 31% GC content chromosome. After making libraries of the resultant amplified genomes, we sequenced the libraries using the 454 Sequencing System [25], and enumerated reads initiating within various sized windows of the respective genome, with a maximum resolution of 10 bases across the entire length of the genome. We then conducted sequence-based karyotyping on the amplified and control genomes, and were thus able to generate a high-resolution comparison, encompassing both coding and non-coding regions, of the coverage bias induced by WGA methods across complete bacterial genomes.

## Results

### Amplification yield

DNA samples were assayed by UV absorption at 260 nm to determine their concentration after amplification. The amount of input DNA was held constant at 25 ng for all methods. Averaged across both genomes, GenomiPhi generated 16.1 μg of DNA, a 640-fold amplification, Repli-G amplified input DNA 2100 fold to 53.6 μg, PEP generated 3.0 μg, a 120-fold increase, and DOP amplified the input DNA 92-fold to 2.3 μg.

### Data analysis

For each amplification method, sequencing reads that mapped to the target genome at 95% or greater accuracy were pooled from three or more individual sequencing runs. The percentage of sequences that mapped to the genome varied depending on the amplification method utilized: roughly 60% of the unamplified and MDA amplified reads mapped at 95% accuracy or better, while 40% of the PEP and 20% of the DOP samples mapped to their target genomes. The number of pooled, mapped reads for each of the amplified samples exceeded 150,000, while the number of pooled control reads was approximately 1,500,000 for each genome, reflecting the larger number of samples drawn from these pools to assess variability within the controls.

Analysis populations composed of 100,000 unique reads, their start position (in base pairs), read length (in bases) and their orientation (forward or reverse) on the reference genome were randomly sampled from each of the pooled sequences from amplified genomic material. Five separate analysis populations were generated from each of the control sequence pools to determine the degree of variation within the unamplified reads. Following the generation of the analysis populations, the total genome coverage in bases was determined for each population and both genomes, and the percent of total coverage calculated for each in Table 1.

**Table 1:** Comparison of genome coverage. Coverage was derived from the individual sequences generated from either unamplified control or whole genome amplified samples.

*Halobacterium *species NRC-1			
**Sample**	**Total Bases Sequenced**	**Nonredundant Bases Sequenced**	**Percent of Genome**

Unamplified Control	9543911	2183656	84.9%
Unamplified Replicate 1	9540329	2188525	85.1%
Unamplified Replicate 2	9540870	2184161	85.0%
Unamplified Replicate 3	9541768	2179647	84.8%
Unamplified Replicate 4	9543517	2183939	84.9%
Averaged Replicates	9541621	2184068	84.9%
GenomiPhi	9736445	1287564	50.1%
Repli-G	9432145	933686	36.3%
PEP-PCR	9410215	921446	35.8%
DOP-PCR	9010086	249571	9.7%

*Campylobacter jejuni*			

**Sample**	**Total Bases Sequenced**	**Nonredundant Bases Sequenced**	**Percent of Genome**

Unamplified Control	10605551	1635277	99.6%
Unamplified Replicate 1	10605882	1636507	99.7%
Unamplified Replicate 2	10592192	1636033	99.7%
Unamplified Replicate 3	10603419	1636620	99.7%
Unamplified Replicate 4	10588370	1637090	99.7%
Averaged Replicates	10597466	1636563	99.7%
GenomiPhi	10380921	1623858	98.9%
Repli-G	10939177	1625276	99.0%
PEP-PCR	10313911	1549641	94.4%
DOP-PCR	9605188	278645	17.0%

**Figure 1:**
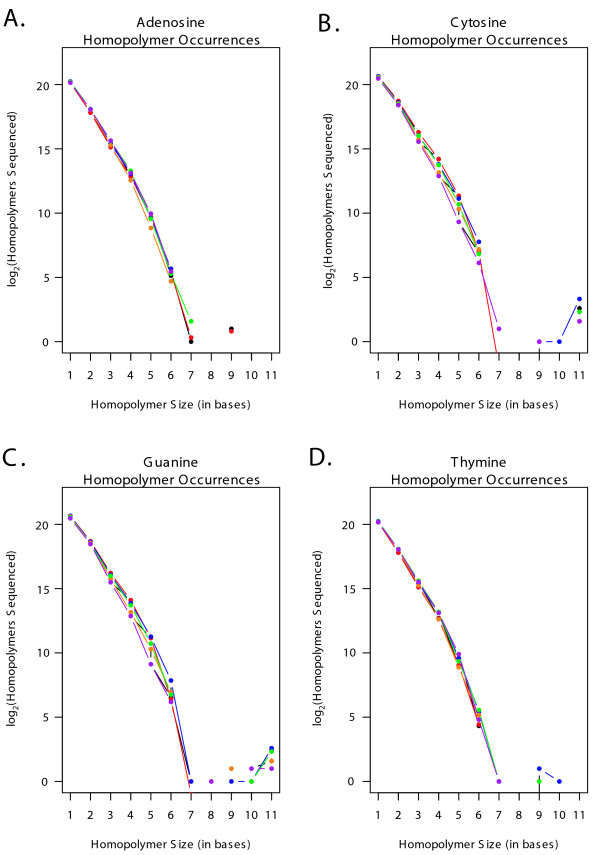
**A – D: Hompolymer coverage in *Halobacterium *sequence reads**. **A**. A log_2_plot illustrating the total number of adenosine homopolymers sequenced in reads generated by control and amplified populations of *Halobacterium *species NRC-1 DNA. The data from the unamplified replicate population are shown in red, GenomiPhi in blue, Repli-G in orange, PEP in green and DOP in purple. **B**. As Figure 1A., but for Cytosine homopolymers. **C**. As Figure 1A., but for Guanine homopolymers. **D**. As Figure 1A., but for Thymine homopolymers.

**Figure 2:**
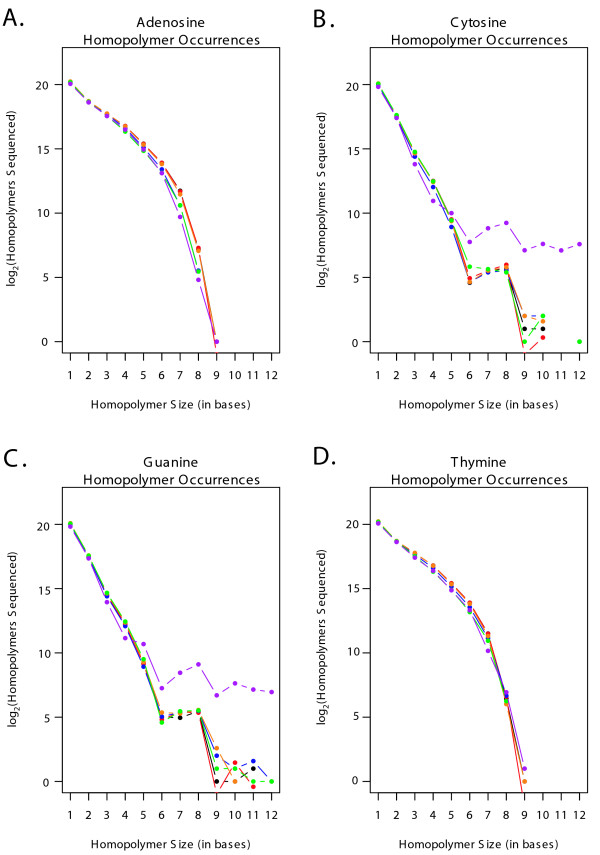
**A – D: Hompolymer coverage in *Campylobacter jejuni *sequence reads**. **A**. A log_2 _plot illustrating the total number of adenosine homopolymers sequenced in reads generated by control and amplified populations of *Campylobacter jejuni *DNA. The data from the unamplified replicate population are shown in red, GenomiPhi in blue, Repli-G in orange, PEP in green and DOP in purple. **B**. As Figure 2A., but for Cytosine homopolymers. **C**. As Figure 2A., but for Guanine homopolymers. **D**. As Figure 2A., but for Thymine homopolymers.

The GC content of the sequenced reads was determined for each genome and amplification methodology. The FASTA files generated for each of the one hundred thousand reads resulting from each amplification method were analyzed for GC content, and the mean GC content and standard deviation for each method was calculated. Welsh's two sample t-test was employed to compare the mean GC content from each test population against the reference population for each genome. The 95% confidence interval around the difference between the means, and the corresponding P-value were recorded in Tables 2 and 3. The sequencing results for both genomes were also analyzed for the type and size of homopolymers covered in the sequencing and summarized in Figures 1 and 2.

**Table 2:** Comparison of *Halobacterium *GC coverage. Mean sequence GC contents, standard deviations, P-values and 95% Confidence limits around differences between the test and unamplified control population means for *Halobacterium species NRC-1*.

Population	P Value	Unamplified Control Mean GC Content	Test Population Mean GC Content	Lower 95% Confidence Limit	Upper 95% Confidence Limit
Unamplified Replicate 1	0.32	61.21 ± 8.6%	61.25 ± 8.6%	-0.11%	0.04%
Unamplified Replicate 2	0.88		61.21 ± 8.6%	-0.07%	0.08%
Unamplified Replicate 3	0.92		61.21 ± 8.6%	-0.07%	0.08%
Unamplified Replicate 4	0.23		61.26 ± 8.6%	-0.12%	0.03%
GenomiPhi	< 0.001		58.29 ± 7.7%	2.85%	2.99%
Repli-G	< 0.001		57.36 ± 7.4%	3.78%	3.92%
PEP-PCR	< 0.001		56.25 ± 7.2%	4.90%	5.04%
DOP-PCR	< 0.001		55.20 ± 7.0%	5.94%	6.08%

**Table 3:** Comparison of *Campylobacter jejuni *GC coverage. Mean sequence GC contents, standard deviations, P-values and 95% Confidence limits around differences between the test and unamplified reference population means for *Campylobacter jejuni*.

Population	P Value	Unamplified Control Mean GC Content	Test Population Mean GC Content	Lower 95% Confidence Limit	Upper 95% Confidence Limit
Unamplified Replicate 1	0.65	31.15 ± 6.7%	31.16 ± 6.7%	-0.07%	0.05%
Unamplified Replicate 2	0.75		31.16 ± 6.7%	-0.07%	0.05%
Unamplified Replicate 3	0.83		31.15 ± 6.7%	-0.07%	0.05%
Unamplified Replicate 4	0.75		31.16 ± 6.7%	-0.07%	0.05%
GenomiPhi	< 0.001		31.36 ± 6.8%	-0.28%	-0.16%
Repli-G	< 0.001		31.60 ± 6.7%	-0.52%	-0.40%
PEP-PCR	< 0.001		33.92 ± 8.1%	-2.84%	-2.71%
DOP-PCR	< 0.001		30.79 ± 5.9%	0.30%	0.41%

**Figure 3:**
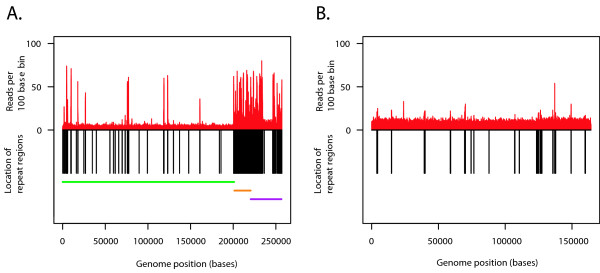
**A & B: Comparison of control sequence coverage versus repeat region location**. Distribution of sequence coverage from unamplified control *Halobacterium *species NRC-1 population as determined by sequence based karyotyping at 100 base resolution relative to repeat region and chromosome location. **A**. Counts per bin are displayed above the X axis in red, repeat regions are shown below the X axis in black. The X axis displays the length of the genome in bases. The relative location of the three *Halobacterium *chromosomes are shown by the horizontal bars below the X axis, NRC1 is green, pNRC100 is gold, and pNRC200 is purple. **B**. As for Figure 3A, but for *Campylobacter jejuni*. No chromosome bars are included as *C. jejuni *is comprised of a single chromosome.

The reference genome was then subdivided into bins of a specific number of bases, and the number of reads that started in each bin was recorded for every population. Additionally, the relationship between coverage depth and the presence of minichromosomes or genomic repeat regions was examined for 100 base genomic bins (See Table 4.). For the purposes of this paper and this sequencing technology, genomic repeats were defined as regions which were 95% identical across 100 bases. The genomic location of the repeat regions relative to the counts per 100 base bins from unamplified *Halobacterium *and *C. jejuni *controls is shown in Figure 3A and 3B.

**Table 4:** Comparison of chromosomal coverage. Sequence coverage per 100 base bin is shown for both repeat and unique regions from unamplified control or whole genome amplified samples.

*Halobacterium *species NRC-1 main (NRC1) and minichromomes (pNRC100 and pNRC200)								
**Chromosome**	**Sample**	**Reads per unique bin**	**Reads per repeat bin**	**Percent chromosomal reads/genome**	**Percent unique chromosomal reads/genome**	**Percent repeat chromosomal reads/genome**	**Percent unique reads/chromosomal reads**	**Percent repeat reads/chromosomal reads**

NRC1 2.01 Mb 68% GC 1.4% repeats	Control	1.91	22.38	44%	38%	6%	86%	14%
	Average Unamplified Replicates	1.92	22.18	44%	38%	6%	86%	14%
	GenomiPhi	0.83	30.92	25%	16%	9%	66%	35%
	Repli-G	0.55	36.23	21%	11%	10%	52%	48%
	PEP-PCR	0.69	28.67	22%	14%	8%	63%	37%
	DOP-PCR	0.38	28.07	15%	8%	8%	49%	51%
pNRC100 191Kb 57.9% GC 79.4% repeats	Control	6.42	13.84	24%	3%	21%	11%	89%
	Average Unamplified Replicates	6.51	13.77	23%	3%	21%	11%	89%
	GenomiPhi	9.47	18.64	32%	4%	28%	12%	88%
	Repli-G	9.81	19.68	34%	4%	30%	12%	88%
	PEP-PCR	11.09	20.51	35%	4%	31%	13%	88%
	DOP-PCR	2.59	20.19	32%	1%	31%	3%	97%
pNRC200 365 Kb 59.2% GC 45.0% repeats	Control	3.78	14.97	32%	8%	25%	24%	76%
	Average Unamplified Replicates	3.85	14.89	32%	8%	24%	24%	76%
	GenomiPhi	4.86	20.19	43%	10%	33%	23%	77%
	Repli-G	4.54	21.92	45%	9%	36%	20%	80%
	PEP-PCR	3.76	21.40	43%	8%	35%	18%	82%
	DOP-PCR	7.65	22.92	53%	15%	38%	29%	71%

*Campylobacter jejuni *single chromosome								

**Chromosome**	**Sample**	**Reads per unique bin**	**Reads per repeat bin**	**Percent chromosomal reads/genome**	**Percent unique chromosomal reads/genome**	**Percent repeat chromosomal reads/genome**	**Percent unique reads/chromosomal reads**	**Percent repeat reads/chromosomal reads**

C. jejuni 1.64 Mb 31% GC 2.71% repeat	Control	5.90	12.94	N/A	94%	6%	N/A	N/A
	Average Unamplified Replicates	5.90	12.97		94%	6%		
	GenomiPhi	5.96	10.81		95%	5%		
	Repli-G	5.87	13.85		94%	6%		
	PEP-PCR	5.35	32.42		86%	15%		
	DOP-PCR	6.12	4.91		98%	2%		

For each sample, the number of reads initiated in each bin was compared to the number found in the same bin of the respective unamplified control. The ratio of the two numbers was computed for each bin, and the maximum (representing over-amplification relative to the control) and minimum ratio (representing under-amplification or sequence loss relative to the control) were recorded in Table 5. As an identical number of reads were used for each sample, the average numbers of reads obtained per bin were identical for each treatment, requiring a more sophisticated statistical assessment to accurately assess potential bias.

**Table 5:** Raw differences in counts per bin between control and test populations. Numbers in parenthesis are the ratio of the WGA amplified samples normalized to the average deviation in the unamplified replicate populations.

**Halobacterium – 100 base bins**								
Versus Unamplified Control	Replicate 1	Replicate 2	Replicate 3	Replicate 4	GenomiPhi	Repli-G	PEP-PCR	DOP-PCR
Maximum Fold Overamplification	10	10	14	11	43 (3.8)	69 (6.1)	299 (26.6)	1633 (145.2)
Maximum Fold Underamplification	11	12	10	11	17 (1.5)	17 (1.5)	24 (2.2)	74 (6.7)
Maximum Difference in Counts per bin	27	26	31	35	67	101	299	1633

**Campylobacter – 100 base bins**								

Versus Unamplified Control	Replicate 1	Replicate 2	Replicate 3	Replicate 4	GenomiPhi	Repli-G	PEP-PCR	DOP-PCR
Maximum Fold Overamplification	13	13	13	14	16 (1.2)	23 (1.7)	31 (2.3)	2668.5 (201.4)
Maximum Fold Underamplification	11	15	12	14	15 (1.2)	16 (1.2)	19 (1.5)	54 (4.2)
Maximum Difference in Counts per bin	18	17	15	17	23	23	6251	133

**Halobacterium – 10 base bins**								

Versus Unamplified Control	Replicate 1	Replicate 2	Replicate 3	Replicate 4	GenomiPhi	Repli-G	PEP-PCR	DOP-PCR
Maximum Fold Overamplification	11	11	11	11	26 (2.4)	23 (2.1)	52 (4.7)	458 (41.6)
Maximum Fold Underamplification	10	9	9	12	15 (1.5)	15 (1.5)	15 (1.5)	15 (1.5)
Maximum Difference in Counts per bin	10	12	12	12	25	22	51	457

**Campylobacter – 10 base bins**								

Versus Unamplified Control	Replicate 1	Replicate 2	Replicate 3	Replicate 4	GenomiPhi	Repli-G	PEP-PCR	DOP-PCR
Maximum Fold Overamplification	7	9	7	8	9 (1.2)	14 (1.8)	39 (5.0)	991 (127.9)
Maximum Fold Underamplification	8	7	8	7	11 (1.5)	11 (1.5)	11 (1.5)	11 (1.5)
Maximum Difference in Counts per bin	8	8	7	7	10	13	38	990

**Figure 4:**
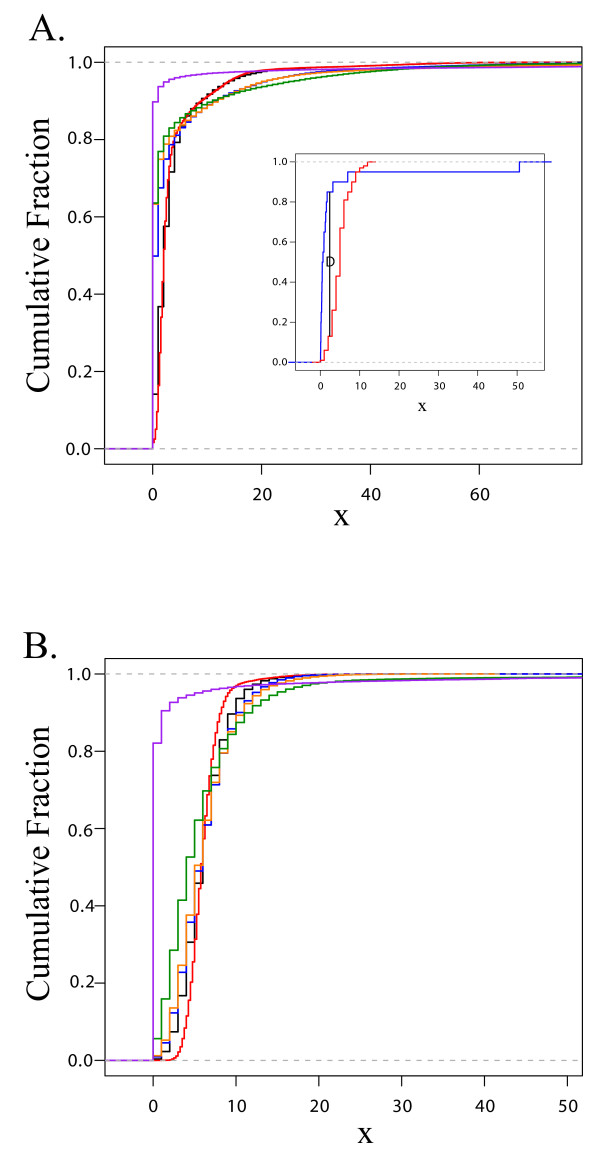
**A & B: Empirical cumulative distribution functions**. **A**. Empirical cumulative distribution function (ECDF) depicting the distributions of counts per bin for various control and amplified populations of *Halobacterium *species NRC-1 DNA. The ECDF represents the cumulative distribution of the number of counts per bin, reporting the cumulative proportion of bins with counts equal or less than the value on the X axis. The control cumulative fraction (black) is plotted against all WGA approaches; GenomiPhi in blue, Repli-G in orange, PEP in green and DOP in purple. **Inset**. Generic ECDF of two different distributions (red and blue), the D statistic is shown as black vertical line labelled "D". **B **as Figure 4A, but derived from *Campylobacter jejuni*.

The empirical cumulative frequency distribution (ECDF) of the reads per bin for *Halobacterium *and *C. jejuni *are shown in Figures 4A and 4B respectively. The ECDF represents the cumulative distribution of the number of counts per bin, reporting the cumulative proportion of bins with counts equal or less than the value on the X axis. It was expected that the counts per bin would follow a Poisson distribution, and some bins in each sample would doubtlessly contain outliers. To address relative bias we wanted to compare differences between the read distributions obtained for each sample, rather than comparing each sample to a model distribution. As a result, the non-parametric, distribution-free Kolmogorov-Smirnov test (KS-test) with its associated D-statistic was used for subsequent analysis.

### The Kolmogorov-Smirnov test (KS-test) and D-statistic

The KS-test was employed to assess the deviation between the number of counts per bin that occurred between two genomes (one test, one reference), using the maximal deviation (D-statistic) between the cumulative count distributions of the populations being compared (for a visual demonstration of the D-statistic, please see the inset on Figure 4A). The D-statistic for each comparison reported the maximum deviation between each sample's cumulative distribution of counts per bin across the entire genome, revealing the greatest extent of distortion or bias relative to the reference distribution. By comparing several test distributions (or genomes) to a single reference, the relative amount of deviation or bias could be determined.

For each genome in this study, the distributions of reads per bin were compared between the five unamplified controls with a KS-test to test for significant variation within the control group. One of the unamplified populations was selected at random to serve as a control, and the distribution of counts per bin from the other four unamplified samples were compared to this. In every case no significant difference (P values ≥ 0.68) was discovered between the control and unamplified replicate populations (please see Additional file 1, Tables S1 and S2). For subsequent comparisons unamplified replicate #3 was chosen (at random) as the control against which the amplified distributions were compared. The D-statistics associated with the control and amplified genomes were then ranked from lowest to highest creating a hierarchy of amplification bias relative to the unamplified reference population.

**Figure 5:**
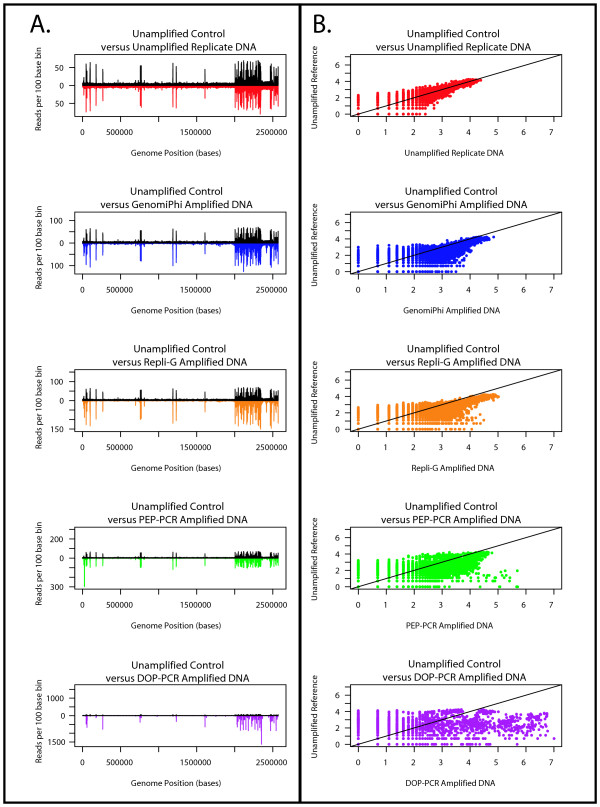
**A & B: Comparison of *Halobacterium *sequence coverage**. Distribution of sequence coverage from unamplified reference, unamplified replicate and whole genome amplified *Halobacterium *species NRC-1 populations as determined by sequence based karyotyping at 100 base resolution. **A**. Distribution of the counts per bin (y-axis) across the length of the genome (X axis) of test populations relative to the unamplified reference population. The counts per bin from the unamplified reference population are depicted in black above the X axis, counts from the various test populations are inverted and shown below the X axis. The data from the unamplified replicate population is shown in red, GenomiPhi in blue, Repli-G in orange, PEP in green and DOP in purple. **B**. Log-log plot of the counts per bin from the unamplified reference population versus the various test populations. The comparison between the reference and the unamplified replicate population is shown in red, GenomiPhi in blue, Repli-G in orange, PEP in green and DOP in purple. A 45 degree black line is shown for comparison.

**Figure 6:**
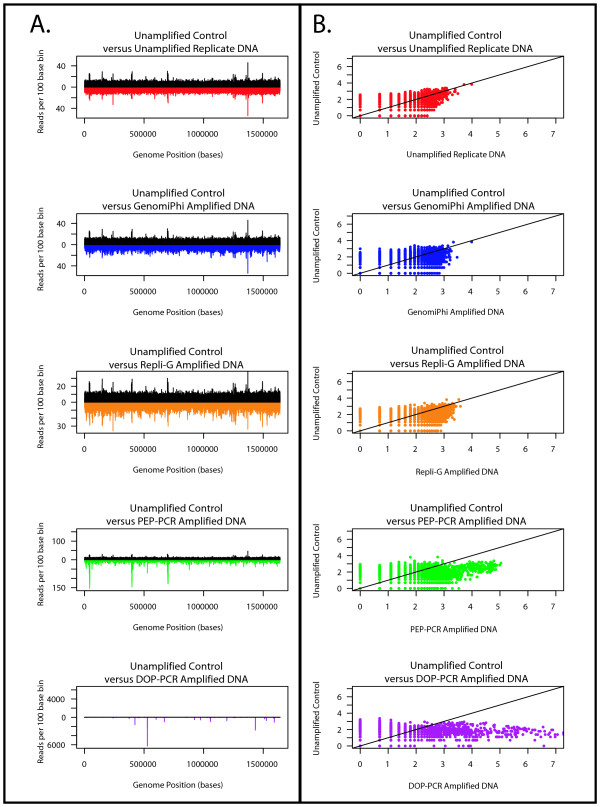
**A & B: Comparison of *Campylobacter *sequence coverage**. Distribution of sequence coverage from unamplified reference, unamplified replicate and whole genome amplified *Campylobacter jejuni *populations as determined by sequence based karyotyping at 100 base resolution. **A**. Distribution of the counts per bin (y-axis) across the length of the genome (X axis) of test populations relative to the unamplified reference population. The counts per bin from the unamplified reference population are depicted in black above the X axis, counts from the various test populations are inverted and shown below the X axis. The data from the unamplified replicate population is shown in red, GenomiPhi in blue, Repli-G in orange, PEP in green and DOP in purple. **B**. Log-log plot of the counts per bin from the unamplified reference population versus the various test populations. The comparison between the reference and the unamplified replicate population is shown in red, GenomiPhi in blue, Repli-G in orange, PEP in green and DOP in purple. A 45 degree black line is shown for comparison.

To ensure that the trends were consistent, the bin size for each genome was increased, effectively decreasing the number of bins and increasing the number of reads per bin, and the process repeated. Four different bin sizes, ranging from 10 bases to 1/100^th ^of the full genome size were used for each organism; the results for *Halobacterium *species NRC-1 and *Campylobacter jejuni *are summarized in Tables 6 &7. A graphical summary of the number of reads per 100 base bin for the different sample populations relative to the unamplified control genome can be seen in Figure 5A for *Halobacterium *and Figure 6A for *C. jejuni*.

**Table 6:** Comparison of *Halobacterium *coverage bias. Kolmogorov-Smirnov comparison of the distributions of reads per bin from an unamplified sample of *Halobacterium *species NRC-1 with an additional unamplified replicate library and libraries amplified with GenomiPhi, Repli-G, PEP and DOP. Bin Size refers to the number of bases comprising each individual bin into which the genome was broken for analysis; 100,000 reads were used for each analysis. Ranked bias was derived from ranked D statistics, lowest to highest.

**Unamplified Control versus:**	**Unamplified Replicate**	**GenomiPhi**	**RepliG**	**PEP**	**DOP**
**Bin Size (bp)**	10				
**Number of Bins**	257102				
**Number of Reads**	100000				
**D Statistic**	0.001	0.081	0.099	0.108	0.213
**P value**	1.000	0.000	0.000	0.000	0.000
**Ranked Bias (5 is lowest)**	5	4	3	2	1
					
**Bin Size (bp)**	100				
**Number of Bins**	25711				
**Number of Reads**	100000				
**D Statistic**	0.003	0.357	0.492	0.495	0.756
**P value**	0.980	0.000	0.000	0.000	0.000
**Ranked Bias (5 is lowest)**	5	4	3	2	1
					
**Bin Size (bp)**	2571				
**Number of Bins**	1001				
**Number of Reads**	100000				
**D Statistic**	0.028	0.648	0.717	0.736	0.837
**P value**	0.791	0.000	0.000	0.000	0.000
**Ranked Bias (5 is lowest)**	5	4	3	2	1
					
**Bin Size (bp)**	25711				
**Number of Bins**	100				
**Number of Reads**	100000				
**D Statistic**	0.095	0.630	0.650	0.660	0.720
**P value**	0.737	0.000	0.000	0.000	0.000
**Ranked Bias (5 is lowest)**	5	4	3	2	1

**Table 7:** Comparison of *Campylobacter *coverage bias. Kolmogorov-Smirnov comparison of the distributions of reads per bin from an unamplified sample of *Campylobacter jejuni *with an additional unamplified replicate library and libraries amplified with GenomiPhi, Repli-G, PEP and DOP. Bin Size refers to the number of bases comprising each individual bin into which the genome was broken for analysis; 100,000 reads were used for each analysis. Ranked bias was derived from ranked D statistics, lowest to highest.

**Unamplified Control versus:**	**Unamplified Replicate**	**GenomiPhi**	**RepliG**	**PEP**	**DOP**
**Bin Size (bp)**	10				
**Number of Bins**	164149				
**Number of Reads**	100000				
**D Statistic**	0.001	0.034	0.029	0.122	0.392
**P value**	1.000	0.000	0.000	0.000	0.000
**Ranked Bias (5 is lowest)**	5	3	4	2	1
					
**Bin Size (bp)**	100				
**Number of Bins**	16415				
**Number of Reads**	100000				
**D Statistic**	0.004	0.060	0.079	0.247	0.882
**P value**	0.997	0.000	0.000	0.000	0.000
**Ranked Bias (5 is lowest)**	5	4	3	2	1
					
**Bin Size (bp)**	1641				
**Number of Bins**	1001				
**Number of Reads**	100000				
**D Statistic**	0.028	0.214	0.275	0.483	0.915
**P value**	0.814	0.000	0.000	0.000	0.000
**Ranked Bias (5 is lowest)**	5	4	3	2	1
					
**Bin Size (bp)**	16413				
**Number of Bins**	100				
**Number of Reads**	100000				
**D Statistic**	0.074	0.248	0.307	0.505	0.842
**P value**	0.922	0.004	0.000	0.000	0.000
**Ranked Bias (5 is lowest)**	5	4	3	2	1

## Discussion

### Amplification yield

The yield of the various whole genome amplification techniques, averaged across both genomes, shows clear differences in the amount of DNA generated by the different methodologies. Although other methods exist (PicoGreen, etc), we chose UV spectrophotometry quantification due to the need to detect both single and double stranded DNA, and the fact that all of the DNA had been purified prior to quantitation, thereby removing the excess nucleotides and primers. It is theoretically possible that some of the hexamer primers may have carried through the sodium acetate precipitation step used in the MDA reactions. However, we do not feel that this unduly influenced the yield reported for the MDA reactions as the OD_260 _method was suggested in the GenomiPhi product manual, and the MDA yields we produced were not excessive.

Each reaction started with 25 ng of input DNA, Repli-G generated a 2100 fold amplification of the input DNA, GenomiPhi 640-fold, iPEP 120-fold and DOP amplified the starting material 92-fold. Sharp contrasts are evident between the yields derived from MDA-based (GenomiPhi and Repli-G) amplifications versus PCR-based (iPEP and DOP) approaches; these can be attributed to the differences between the highly processive strand displacement activities of ϕ29, the polymerase used in both MDA reactions, and the Taq-like enzymes used in the PCR based reactions. Due to the strand displacing capabilities of ϕ29, MDA reactions do not require repetitive cycles of denaturation and annealing temperatures. In PCR reactions these cycles limit polymerase longevity and activity; the half-life for various Taq polymerases ranges from 30 to 70 minutes at 95°C, resulting in a 50% or greater decrease in enzyme activity at the end of 40 cycles [26]. By utilizing isothermal reactions the MDA methods are able to preserve enzyme functionality for a full 16 hour reaction, and generate substantially more DNA in the process by a hyperbranched mechanism [27] of DNA amplification. It is interesting to note the difference in yield between the MDA methods; it is likely that the use of the KOH alkali denaturation prior to amplification used in the Repli-G process is more efficient at opening potential priming sites than the thermal denaturation used in the GenomiPhi protocol. This is in general agreement with findings that the quality of Sanger-sequence data from MDA-amplified templates improved after alkali denaturation [23], and increased PCR yields following NaOH, as opposed to thermal, denaturation of a high GC genome [28].

### Genome coverage

The percent of genome coverage varied considerably by genome and amplification methodology (See Table 1). The unamplified samples, both reference and all unamplified replicates, covered similar expanses of the target genomes, obtaining 84.9 to 85.1% coverage of *Halobacterium *species NRC-1 and 99.6 to 99.7% of *Campylobacter jejuni*. As the *Halobacterium *species NRC-1 genome is 1.54 times larger than *Campylobacter jejuni *(2.56 versus 1.64 MB) it is not surprising that 100,000 reads complete relatively less of the larger *Halobacterium *sequence. However, the substantial difference between the 85% completion of *Halobacterium *and the 99% *Campylobacter jejuni *completion obtained from the unamplified samples requires additional explanation. Analysis of the number of reads per *Halobacterium *chromosome reveals that the number of reads per 100 base bin is disproportionately high for both pNRC100 (12 reads/bin on average) and pNRC200 (8.8 reads per bin) than for the main chromosome NRC1 (an average of 2.2 reads per bin) (See Table 4.). The over-representation of the minichromosome reads even in the unamplified samples may simply reflect a greater abundance of the smaller chromosomes relative to the main chromosome; pNRC100 is a multicopy chromosome [24], and the same might be true for pNRC200. Additionally, a significantly higher percentage of the *Halobacterium *genome is comprised of repeat regions (13.37% versus 2.71% repeat for *C. jejuni*), and the smaller *Halobacterium *chromosomes contain a higher incidence of repeats than the main chromosome (1.4%, 79.4% and 45% repeat for NRC1, pNRC100 and pNRC200 respectively). The repeat regions are disproportionately heavily sequenced relative to their frequency, encompassing 14%, 89% and 75% of the control reads from NRC1, pNRC100 and pNRC200 respectively (Table 4., Figures 3A and 3B). The relative over-coverage of the repeat regions results in fewer of the 100,000 reads covering unique regions, and thus reduces the total coverage of the *Halobacterium *species NRC-1 genome. *Campylobacter jejuni *also experiences relative oversampling of the repeat regions, but with far fewer repeat regions and a smaller total genome than *Halobacterium*, total genome coverage is less affected.

Total coverage of *Halobacterium *dropped significantly when amplified DNA was sequenced, with coverage ranging from 50.1 to 9.7% depending on the amplification process. *C. jejuni *coverage was more complete for most of the amplified material; GenomiPhi, Repli-G and PEP-PCR amplified samples covered 98.9, 99.0 and 94.4% of the genome respectively, while DOP-PCR only covered 17%. The relatively poor coverage of *Halobacterium *by the WGA methods may be due to the presence of the two minichromosomes, and the frequency of repeat regions within them. As mentioned previously, the minichromosomes are more repeat-rich than the main chromosome, there may be relatively more copies of the minichromosomes present in the population relative to the main chromosome, and the smaller chromosomes are more likely than the larger main chromosome to have survived the DNA purification process and shipment intact. Coupled with the high incidence of repeat sequences in the minichromosomes, this could cause a disproportionate amplification of the two minichromosomes, and corresponding lower sequence coverage of the remaining chromosome.

Additionally, as the two minichromosomes have lower GC contents than the main chromosome (57.9 and 59.2% for pNRC100 and pNRC200 respectively, relative to 67% for the main chromosome); the possibility of preferential, GC-influenced amplification was investigated. For either genome the mean sequence GC content was not significantly different (p > 0.05) between the unamplified control and the replicate populations. The control values for *C. jejuni *were in close agreement with the listed GC content (31% versus 31.15%), while the values obtained for *Halobacterium species NRC-1 *were slightly lower than the listed values (61.2% versus 66%), possibly reflecting both the lower proportional coverage of the genome, as well as inherent difficulty in sequencing strands with high degrees of secondary structure.

The mean sequence GC contents for all WGA methodologies were significantly different (p ≤ 0.001) from the control GC content for both genomes. Deviation from the control GC content was less pronounced for *Campylobacter jejuni*, the low GC genome. Sequences generated by GenomiPhi and Repli-G were roughly 0.2% and 0.5% more GC rich than the control, respectively, while PEP-PCR produced reads that were roughly 2.5% more GC rich than the unamplified control. In contrast, DOP-PCR reads were biased in the opposite direction, 0.3 to 0.4% more AT rich than the control sequence.

The sequences produced by each of the WGA methods were significantly lower in GC than the unamplified *Halobacterium species NRC-1 *reference sequence, and the margin of difference was more pronounced than described for *C. jejuni*. GenomiPhi sequences were the closest to the control GC content, with roughly 2.9% lower GC content in the amplified sequence. Repli-G was the next highest, with roughly 3.8% less GC than the control. The two PCR-based methods generated the most biased sequence in terms of GC content: the mean GC content of PEP-PCR reads were between 4.9 and 5.0% lower than the control, while DOP-PCR reads contained 5.9 to 6.08% less GC on average.

The relative abundance and size of homopolymer stretches in the target genome could possibly influence genome coverage through polymerase slippage in whole genome amplified samples. The frequency of A, C, T and G homopolymers were calculated separately for each genome and sample. The data were log_2 _transformed to allow an informative scale and resolution and shown in Figures 1 and 2 for *Halobacterium *and *C. jejuni *respectively. While a thorough analysis of the influence of template composition on sequence coverage and amplification is beyond the scope of this paper, the figures illustrate that homopolymer coverage is similar for most samples across all four nucleotides and both genomes. One exception is found in the DOP-PCR coverage of C and G homopolymers in *C. jejuni *(purple data points in Figure 2B and 2C), where the frequency of the G and C homopolymers is substantially greater than those in the controls and other amplified samples for homopolymers from 6 to 12 nucleotides long.

Sequence coverage bias related to MDA has been reported in the past. Dean *et al*. [29] detected preferential amplification of pUC19 from samples containing both plasmid and bacterial chromosomal DNA. In their case, however, the pUC19 plasmid was only 2.7 kb in circumference, and thus easily circumnavigated by a single ϕ29 molecule, which generates an average product of 10 kb or greater [15]. In our study, however, the size difference between the main and mini *Halobacterium species NRC-1 *chromosomes is less dramatic, and all chromosomes exceed 100 kb in length, rendering it unlikely that polymerase processivity is the root cause of the increased minichromosomes coverage. It is possible, as suggested by Dean *et al*. [29] that the smaller chromosomes are less likely to suffer nicking and subsequent RCA termination than larger chromosomes. As mentioned previously, Paez *et al*. [23] reported selective under-representation and loss from amplified material, Raghunathan *et al*. [9]described bias from 0.1% to 1211% in a qPCR analysis of MDA from single *E. coli *cells, sequence loss related to regional proximity to the ends of both human and yeast chromosomes has also been detected [16], and MDA-induced loci over and under representation as high as 6 fold has been discovered by qPCR [20]. MDA amplification of multiple bacterial species within a single sample revealed preferential amplification of 3 of the 8 species, although all species were represented to some extent [30]. In this study, differences in template size, stochastic effects and other factors were thought to be influential [30].

It is interesting to note that in our study none of the previously mentioned possibilities (repeat regions, chromosome size and GC content) can fully explain the over-amplification of the mini-chromosomes. Should these factors directly influence the relative amplification rate, one would expect pNRC100 to display a higher amplification rate than pNRC200 as pNRC100 is smaller, has a lower GC content, and is substantially more repeat rich (Table 4.). This is not the case; however, as all WGA samples provide higher coverage for pNRC200 than they do for either pNRC1 or pNRC100. As a result, it is impossible to precisely attribute the cause of the bias as uniquely size, copy number, repeat, homopolymer or GC derived, and overamplification appears to be influenced by factors other than those we have discussed here.

### Internal controls and run to run reproducibility

Unamplified *Halobacterium *and *C. jejuni *DNA were made into libraries and sequenced as controls both for comparison to the WGA samples and to ensure that any potential systemic bias introduced by the sequencing platform was reproducible from sample to sample. Comparisons between all five unamplified populations from both genomes displayed similar and low count deviation between identical bins (Table 5.) and returned non-significant P-values (P values ≥ 0.68) at all bin sizes (Tables S1 and S2), with similar non-significant P-values between the unamplified reference sample and the unamplified control (See Tables 6 and 7). Similarly, reads per bin from the control plotted against the reference displayed similar location and magnitude of over-represented reads (Figures 5A and 6A, red/black plots), with the previously mentioned over-representation of repeat regions and the smaller minichromosomes (Figures 3A and 3B). Log-log plots of the reads per bin (Figures 5B and 6B, red plots) reveal a strongly linear relationship that indicates that multiple sequencing runs do not induce significant bias in samples generated from the same original genomic library. Having established the unamplified DNA libraries as a baseline, potential bias in the whole genome amplified libraries was quantified.

### Bias ranking whole genome amplified template DNA

Regardless of bin size and genome, all whole genome amplified samples displayed significant bias relative to the unamplified reference (See Tables 6 and 7). With one exception the relative amount of bias induced by the various methods remained consistent across both genomes and all bin size resolutions, with GenomiPhi generating the lowest D-statistics, followed by Repli-G, PEP and DOP. The exception was found in the 10 base resolution analysis for *C. jejuni*, where GenomiPhi and Repli-G exchanged position; while significant, the difference between the Repli-G and GenomiPhi samples in this instance was slight (0.005). Increased bin size elevated the magnitude of the D-statistics for all test and control populations, although the unamplified control D-statistics increased more rapidly with bin size than did the test populations (Table 8), effectively decreasing the bias as bin size increased. This, however, is merely the result of smoothing a constant number of reads per population across a diminishing number of bins, and does not reflect any amplification-specific size related effect.

**Table 8:** WGA-induced bias relative to control populations. KS-test D-statistic ratios relative to the unamplified replicate at increasing bin sizes.

*Halobacterium *species NRC-1				
**Bin Size (bases)**	**GenomiPhi**	**RepliG**	**PEP**	**DOP**

10	81.0	99.0	108.0	213.0
100	119.0	164.0	165.0	252.0
2571	23.1	25.6	26.3	29.9
25711	6.6	6.8	7.0	7.6

				
*Campylobacter jejuni*				

**Bin Size (bases)**	**GenomiPhi**	**RepliG**	**PEP**	**DOP**

10	34.0	29.0	122.0	392.0
100	15.0	19.8	61.8	220.5
1641	7.6	9.8	17.2	32.7
16413	3.4	4.2	6.8	11.4

The deviation in counts per bin (Table 5) and the D-statistics were 2- to 10-fold lower for *C. jejuni *than for *Halobacterium *for all amplified populations, although the biases between the amplified *C. jejuni *populations were more extreme (Table 9). This may reflect the fact that *C. jejuni*, a smaller circular genome, is better suited to the isothermal, highly processive MDA approaches than the cyclically denatured, less-processive PCR-based methods. Similar to the genome coverage results, the D-statistics indicated that the presence of mini-chromosomes and/or high GC content in *Halobacterium *challenged all amplification techniques and reduced the advantage from the MDA process.

**Table 9:** Maximum and minimum bias relative to control populations. Minimum and maximum fold differences in KS-test D-statistic values between amplified samples for all bin sizes.

*Halobacterium *species NRC-1			
	**Repli-G**	**PEP**	**DOP**

**GenomiPhi**	1.03 to 1.37	1.04 to 1.38	1.14 to 2.62
**Repli-G**		1.01 to 1.09	1.11 to 2.15
**PEP**			1.09 to 1.98

*Campylobacter jejuni*			

	**Repli-G**	**PEP**	**DOP**

**GenomiPhi**	0.85 to 1.30	2.04 to 4.09	3.4 to 14.58
**Repli-G**		1.64 to 4.28	2.74 to 13.7
**PEP**			1.67 to 3.57

### Multiple displacement amplification methods

The two Multiple Displacement Amplification (MDA) methods, GenomiPhi and Repli-G, introduced the lowest amplification bias, inducing the least distortion in counts per bin across the length of the genome and slight decreases in slope below the 45 degree line in the log-log plot (Figures 5 and 6, blue and orange plots respectively). The maximum deviation from the reference distribution at 10 base resolution was 81 and 34 fold greater than the unamplified control for GenomiPhi on *Halobacterium *and *C. jejuni *respectively, and 99 and 29 fold greater than *Halobacterium *and *C. jejuni *control for Repli-G (Table 8). Differences between the MDA methods were slight, particularly in light of the aforementioned reversal in bias rank in the *C. jejuni *10 base bin analysis. Regardless of bin size, the D-statistic for Repli-G was 1.03 to 1.37 times larger than that from GenomiPhi for *Halobacterium *and 0.85 to 1.30 times greater for *C. jejuni *(Table 9).

The bias estimates we derived for MDA are comparable to those reported in the literature. The crude metric of counts per bin (Table 5) yields estimates of 2.4 (10 base bin) to 3.8 fold (100 base bin) overamplification relative to the average counts per bin for the unamplified replicates for *Halobacterium *when GenomiPhi was used, and 2.1 to 6.1 fold for Repli-G. Raw count bias in *C. jejuni *was 1.2 fold for both bin sizes for GenomiPhi and 1.7 (100 base bins) to 1.8 (10 base bins) for Repli-G. The biases estimated for each genome by D-statistic are smaller but still comparable to the 0.5- to 3-fold biases reported by Dean *et al*. [15] and Hosono *et al*. [20] as well as the 0.75 to 1.35-fold biases for multi-cell samples in Raghunathan's study [9]. This is particularly interesting as the studies relied upon gene locus sequencing or qPCR quantitation, which focuses exclusively on coding regions of the genome, while our whole genome sequencing included both coding and non-coding regions, yet still reached similar estimates of bias.

### PCR-based amplification methods

PCR-based amplification methods were more biased than MDA methods for all bin sizes and both genomes, although the PEP method was less biased than the DOP amplified samples. Visually, the counts per bin are obviously distorted around the repeat regions for PEP amplified material (Figures 5A &6A, green plot), and even more drastically distorted for DOP samples (Figures 5A &6A, purple plot). Log-log plots display a distinct non-linear relationship between reference and amplified samples, with the data cloud shifting to the right for both amplification methods, with more skew evident in DOP than PEP (Figures 5B &6B). At a 10 base resolution, D-statistics from PEP amplified *Halobacterium *and *C. jejuni *were 108 and 122 times greater than the reference value respectively, while D-statistics from DOP amplified material were respectively 213 and 392 times larger than the *Halobacterium *and *C. jejuni *reference. In comparisons between the PCR-based methods, the maximal deviation from the reference for the PEP amplified *Halobacterium *sample was 1.09 to 1.98 times lower than that amplified by DOP; the D-statistic from the *C. jejuni *sample was 1.67 to 3.57 fold lower with PEP relative to DOP (See Table 9.).

Differing results between the PCR-based amplification methods can be partially attributed to the primer composition in each reaction. Both MDA and PEP amplification used random bases (6 bases for MDA, 15 bases for PEP), while DOP employed a 22 base primer containing a random 6-mer flanked with specific 10-mer 5' and 6-mer 3' sequences. As amplification events were initiated from the annealed specific 3' region of the primer, this resulted in preferential amplification or over-amplification of genomic regions that permitted successful primer binding. Similarly, regions where the primer hybridized poorly were amplified infrequently. Additionally, PCR-driven competition for primers, polymerase, etc. may prevent any significant representation of the infrequently primed amplicons in the final amplified pool. These factors resulted in over-representation of genome regions containing the 3' primer sequence, and was clearly evident in the overamplification spike around base position 540,000 (which does not coincide with a repeat region in the unamplified references) in *C. jejuni *genome. In the course of sequencing the DOP libraries, it was noted that sequences containing the DOP 5' specific decamer (5' CCGACTCGAG 3') also contained the exact 3' hexamer (5' ATGTGG 3') 88% of the time, and matched 5 of the 6 bases at the 3' end 95% of the time. Despite the low stringency of the DOP thermocycling protocol, the DOP primer annealed primarily to regions with at least 5 of 6 bases of homology to the 3' end of the DOP primer. The tendency to amplify only those regions flanked by the DOP primer sequence is a significant barrier to the applicability of DOP protocol for amplification of entire genomes.

These findings are in agreement with much of the literature which finds that although DOP-PCR amplified human DNA is sufficient for general genotyping purposes [31], sequence-specific overamplification and subsequent coverage bias results from amplification of smaller size, lower complexity genomes such as cosmids and plasmids [18]. Lower complexity genomes would be expected to have lower, less uniform incidence of the 3' priming sequence than larger genomes, resulting in more bias from the amplified bacterial genomes used our research than the human DNA examined by Cheung *et al*. (1996). Simulated studies of DOP-PCR uniformity found that for a given DOP primer sequence, the specific regions of DOP overamplification could be accurately predicted for several eukaryotic genomes [32], although some (~22%) of the regions expected to amplify in the study failed to generate any product at all, illustrating the additional affect of bias stemming from amplicon amplification efficiency [33]. Dean *et al*. [15] reported a 6-fold bias in coding regions following PCR-based WGA. The increased bias detected by whole genome sequencing in our study might reflect the fact that coding regions have evolved to permit polymerase access and expression, and therefore coding regions may experience less bias than non-coding regions.

Although in our study PEP amplification generated lower bias than DOP, possibly due to increased degeneracy of the PEP primers, both PEP and DOP suffered from potential biases inherent to PCR amplification. PCR-related bias in products amplified from complex mixtures is well documented in the literature, due to factors including differential GC content [34], product reannealing [35] and primer binding energy differences [36]. Moreover, not all amplicons are amenable to PCR amplification [32], resulting in missing sequence, the high-temperature denaturation required to render genomic DNA single-stranded for PCR-based WGA methods can cause cytosine deamination, and homoduplexes can form during the ramp from denaturation to annealing temperature [37]. All of these factors can result in the generation of up to 70% nonspecific amplification artefacts [31] leading to incomplete coverage [38]. This agrees closely with the high percentage of PEP and DOP generated sequences (60 and 80% respectively, as opposed to 40% for unamplified and MDA samples) that failed to map to the reference genomes in our study. This final point is particularly relevant to researchers who are considering the use of WGA amplified material for sequencing. Given the high rate of non-specific or artifactual product formation in the PCR-based WGA methods, the cost of sequencing is increased due to the poor return of sequence data per template. *De novo *sequencing is more drastically affected as the artifactual products might not be removed prior to contig assembly, and thus waste processing time and possibly generate incorrect scaffolds.

## Conclusion

In this paper, we utilized whole genome sequencing and sequence-based karyotyping to assess the bias induced by four methods of whole genome amplification. While the advantages and disadvantages of each WGA method have been discussed extensively in literature [15,16,19,20], previous comparisons between whole genome amplification techniques have used SNP analysis, CGH or FISH analyses to determine induced bias. SNP analysis excels at detecting single base errors, but since SNP analysis typically examines only a small fraction of a given genome, it has the potential to overlook or miss most amplification infidelities. Additionally, as the loci examined by SNP analysis are typically characterized by minimal sequence differentiation, these loci may be relatively immune to amplification bias as their amplification efficiencies would be roughly equivalent. Bias detection via FISH and CGH, on the other hand, is limited to a mapping resolution of approximately 20 Megabases [39,40], is incapable of detecting small scale errors or distortions and has difficulty detecting homozygous deletions [39].

Previously prohibitively expensive and time consuming, whole genome sequencing of entire genomes is now possible with the 454 sequencing system in a matter of days. Using this approach we conducted a high resolution (10 base) examination of amplification-induced bias, detecting statistically significant (P < 0.001) bias in all amplified DNA samples relative to unamplified controls. MDA-based amplification methods generated the highest amplification yield and most complete genome coverage while introducing the least bias of all the amplification methods examined. Of the MDA methods, Repli-G generated 3 to 4-fold more amplified DNA, but introduced marginally more bias than GenomiPhi, and generated significantly lower genome coverage when amplifying *Halobacterium *species NRC-1. It is important to note that both MDA based processes were challenged by the high GC content, multi-copy minichromosome containing samples. This may result from the fact that more of the small circular chromosomes are probably present in the starting sample, are more likely than large chromosomes to survive extraction and purification processes intact, and strand displacement is more difficult in regions with increased GC content.

In summary, we have determined that none of the whole genome amplification methods we investigated are free from bias. Depending upon the intended application, researchers should careful weigh the decision whether or not to use whole genome amplification. PCR-based WGA methods generated roughly an order of magnitude less amplified DNA than the MDA methods, with concomitantly increased bias. In our analysis, the relatively low efficiency and yield and high bias generated by the PCR-based methodologies renders them unsuitable for whole genome sequencing and representative amplification of precious DNA. This is not to say that the PCR-based methods are without merit, merely that based on our results, their use should be restricted to DNA amplification for genotyping or marker identification purposes [41], not uniform genomic amplification for high accuracy whole genome sequencing. MDA-based techniques generated significantly higher yields of the proper (non-artifactual) template, and induced lower, but still significant levels of bias. Samples destined for high resolution copy number studies, or complex populations composed of genomes with diverse sizes and GC contents, such as environmental samples, may experience detectable and possibly unacceptable amplification bias. In contrast, applications such as strain identification or whole genome sequencing of purified samples may either tolerate the inherent bias, or surmount it through additional sequence oversampling. For applications amenable to amplified DNA, our investigation determined that MDA-based methodologies produce the greatest amplification yield with the lowest associated bias.

## Methods

### Template DNA

Bacterial DNA was obtained from two different sources. The *Campylobacter jejuni *was the generous gift from Dr. Jorge Galan (Dept. of Microbial Pathogenesis, Yale University, New Haven CT.). The DNA was subsequently stored at -20°C until used. Ten micrograms of *Halobacterium *species NRC-1 DNA were purchased from ATCC (Order number 700922D, Manassas, VA) and arrived as a lyophilized pellet. The DNA was reconstituted to a concentration of 1 ug/ul in molecular biology grade water (Eppendorf AG, Hamburg, Germany), and stored at -20°C until needed. The genomic DNA served both as template for whole genome amplification methods and as unamplified control DNA. Three microgram aliquots of each bacterial genomic DNA, representing approximately 1.8 × 10^9 ^genomic equivalents of *C. jejuni *and 1.1 × 10^9 ^genomic *Halobacterium *equivalents, were removed for unamplified controls prior to initiation of the various whole genome amplifications. Once aliquotted, the control DNA samples were stored at -20°C until processed as outlined in Sample Preparation.

### Whole genome amplification methods

#### GenomiPhi

One of the two commercially available Multiple Displacement Amplification kits used in this study was the GenomiPhi DNA Amplification Kit (Amersham Pharmacia, Uppsala, Sweden). For the GenomiPhi reactions, 25 ng of genomic DNA (in 2.5 μl) were mixed with 22.5 μl of GenomiPhi sample buffer. This mix was heat denatured at 95°C for three minutes, then cooled on ice. Twenty-seven microliters of GenomiPhi Reaction buffer were then mixed with 3 μl GenomiPhi enzyme, and 25 μl of this mix were added to the denatured genomic DNA. The reaction was subsequently incubated at 30°C for 14 hours, then heat inactivated at 65°C for 10 minutes. The amplified DNA was pelleted and purified by sodium acetate precipitation and resuspended in 25 μl of 10 mM Tris (pH 7.5).

#### Repli-G

The other Multiple Displacement Amplification kit used in this study was the Repli-G Whole Genome Amplification kit (Qiagen Sciences Inc. Germantown, MD). For the Repli-G reactions, 25 ng of genomic material were diluted in TE to a final volume of 2.5 μl. To this was added 2.5 μl of a 1:8 dilution of freshly made Solution A (0.4 M KOH, 10 mM EDTA); the solution was incubated at room temperature after mixing. After three minutes, 5 μl of Stop Solution (1:10 dilution of Repli-G Solution B) were added to the incubated reaction. A solution consisting of 32.4 μl water, 15 μl of Repli-G 4X Mix and 0.6 μl of Repli-G polymerase were mixed, and 40 μl of this solution were added to the 10 μl of denatured, neutralized genomic DNA. The combined solutions were then mixed and incubated at 30°C for 14 hours. The samples were then heat inactivated at 65°C for 10 minutes, and the DNA was pelleted and purified by sodium acetate precipitation and resuspended in 25 μl of 10 mM Tris (pH 7.5).

#### PEP

For the PEP libraries, *C. jejuni *and *Halobacterium *DNA samples were amplified following the improved PEP protocol (iPEP) [4,5] using the Expand High Fidelity PCR System from Roche Applied Science (Basel, Switzerland). Each 50 μl reaction had a final composition of 0.1 mM dNTPs, 50 μg/ml BSA, 2.5 mM MgCl_2_, 16 μM of random 15-mer PEP primers (5' NNN NNN NNN NNN NNN 3') from Integrated DNA Technologies (Coralville, IA), 25 ng genomic DNA, 3.5 units of Expand High Fidelity Polymerase, and 1X Expand High Fidelity Buffer. The samples were thermocycled as follows: 2 minute denaturation at 94°C followed by 50 cycles of a 40 second denaturation at 94°C, 2 minutes at 37°C, 0.1 °C/second ramp to 55°C, 4 minutes at 55°C, and 30 seconds at 68°C. A 7 minute extension at 68°C followed the final cycle. After thermocycling, the DNA was pooled and purified using a PCR clean-up kit from Qiagen (Valencia, CA). The Qiagen PCR clean-up kit recovers fragments from 40 bp to 10 kb, thus permitting removal of unextended primers without the loss of amplified fragments.

#### DOP

For the DOP libraries, *C. jejuni *and *Halobacterium *DNA samples were amplified using the DOP PCR Master Kit from Roche Applied Science. For the reaction, 25 ng of genomic DNA were added to 25 μl DOP PCR Master Mix, 2.5 μl of the supplied 40 μM DOP PCR primer (5' CCG ACT CGA G NNN NNN ATG TGG 3'), with enough water to bring the volume to 50 μl. The manufacturer's instructions were followed for the long thermocycling protocol, except that the reactions were cycled in 50 μl volumes as opposed to the suggested 100 μl volume. The samples were thermocycled as follows 5 minute denaturation at 95°C, followed by 5 cycles of 1 minute at 94°C, 1.5 minutes at 30°C, ramp to 72°C at 0.2°C/second, and 3 minutes at 72°C, then 35 cycles of: 1 minute at 94°C, 1 minute at 62°C, and 2 minutes at 72°C with 14 seconds added to the 72°C step with each cycle, finishing with 7 minutes at 72°C.

### Sample preparation and amplification

All samples of genomic DNA, whether amplified or control, were treated identically after purification, following the protocol outlined in Margulies *et al*. [25]. The process is summarized briefly as follows: 3 μg of DNA from the respective sample were fragmented by a five minute nebulization at 44 psi. The DNA was then purified over MinElute columns following the PCR clean-up protocol from Qiagen. The purified, fragmented DNA was polished with T4 DNA Polymerase and T4 Polynucleotide Kinase (New England Biolabs, Beverly, MA). After a half hour reaction with kinase, the DNA fragments were purified again over MinElute columns, before blunt-end ligation (New England Biolabs Quick Ligation Kit) to 454's proprietary double-stranded DNA adaptors A and B. The double-stranded adaptors have one blunt end and one overhanging end, and the blunt end has a non-phosphorylated 5' end, so that the adaptors do not self-ligate. The overhanging 5' end of the B adaptor is biotinylated. The ligated DNA fragments were then bound to magnetic, Streptavidin-coated beads (Dynal Biotech, Oslo, Norway). Because of the non-phosphorylated 5'adaptor ends, the ligated fragments contain nicks, which were displaced with *Bst *polymerase (New England Biolabs). Single-stranded DNA was melted away from the beads with 0.125 M NaOH and the single-stranded fragments were purified over Qiagen MinElute columns. The resulting purified DNA library was run on an Agilent 2100 BioAnalyzer using a RNA 6000 Pico LabChip^® ^(Palo Alto, CA) to quantitate the single stranded DNA concentration, ranging from 200 to 500 bases in length, with a mean size of 350 bases.

The library was combined with a solution containing emulsion PCR reagents and DNA capture beads (Amersham Biosciences NHS-activated HP sepharose) and amplification was carried out as described elsewhere [25]. After amplification, emulsion breaking, and enrichment of the library beads, the non-covalently bound strand was melted away, a sequencing primer was annealed to the covalently bound, amplified strands, and the primed DNA beads were sequenced on the 454 Sequencing System.

### Mapping

An alignment algorithm developed by 454 Life Sciences was used to map the reads generated by the 454 sequencing system to the GenBank entries either for *Campylobacter jejuni *(gi|15791399|ref|NC_002163.1| Campylobacter jejuni subsp. jejuni NCTC 11168, complete genome, total length 1.64 Mb, 31% GC) or a composite reference sequence composed of *Halobacterium *species NRC-1 (gi|12057215|ref| AE004437|Halobacterium sp. NRC-1 complete genome, chromosome length 2.01 Mb), and the two associated multicopy minichromosomes pNCR100 (gi|10803547|ref| NC_001869| Halobacterium sp. NRC-1 plasmid pNRC100, complete sequence, 191 Kb in length, 57.9% GC) and pNCR200 gi|12057216ref|AE004438|Halobacterium sp. NRC-1 plasmid pNRC200 complete genome, 365 Kb, 59.2% GC content). This alignment technique matches the light signals measured during each nucleotide flow to the signals expected from the reference sequence. The advantage of this technique is that it takes into consideration that the 454 system sequences DNA one mononucleotide repeat stretch as a time – as opposed to traditional approaches that sequence on a nucleotide-by-nucleotide basis.

Acceptable (or "mapped") alignments were distinguished from rejected (or "unmapped") alignments by calculating the alignment score for each sequence. For this study, an alignment was recorded when the signals from the 5'-end of a read agreed with the expected reference sequence with an average logarithmic probability of at least -1.0. The alignment must also have spanned at least the first 50 light signals from the read. Benchmarking has shown this definition is roughly equivalent to a nucleotide-based alignment with 95% identity over at least the first 30 bases of the read (Data not shown).

Reads that aligned to more than one location on the reference genome were ignored to remove ambiguities concerning the location of the genome from which the DNA actually originated.

## Authors' contributions

Both RP and AdW collaborated on the manuscript, and conducted the various methods of whole genome amplification. GJS was responsible for significant amounts of manuscript proofreading and rewriting. MBG was the main source for statistical insight, and ran extensive simulations to suggest which tests should be run for the analysis. KRT conducted all of the DNA sample preparation work, while RNP was responsible for the sequencing. ME and JMR provided invaluable comments and insights into the project, and suggested key directions in which the work should progress. JHL conceived of, and directed the study. All of the nine have reviewed and approved the final manuscript, and have no competing interests to declare.

## Supplementary Material

Additional File 1 **Table S1. Read distributions between unamplified *Halobacterium *samples**. Kolmogorov-Smirnov comparison of the distributions of reads per bin from an unamplified sample of *Halobacterium *species NRC-1 with four replicate unamplified control libraries. Bin Size refers to the number of bases comprising each individual bin into which the genome was broken for analysis; 100,000 reads were used for each analysis. As no significant differences were found between the distributions, ranked bias values (derived from D statistics) were assumed equivalent and not assigned. **Table S2. Read distributions between unamplified *Campylobacter *samples**. Kolmogorov-Smirnov comparison of the distributions of reads per bin from an unamplified sample of *Campylobacter jejuni *with four replicate unamplified control libraries. Bin Size refers to the number of bases comprising each individual bin into which the genome was broken for analysis; 100,000 reads were used for each analysis. As no significant differences were found between the distributions, ranked bias values (derived from D statistics) were assumed equivalent and not assigned. Click here for file
