# Supplementary material for: Assessment of whole genome amplification-induced bias through high-throughput, massively parallel whole genome sequencing
Source: BMC Genomics. 2006 Aug 23;7:216. doi: 10.1186/1471-2164-7-216 (PMC1560136; doi:10.1186/1471-2164-7-216)
Supplement: Additional File 1 — Table S1. Read distributions between unamplified Halobacterium samples. Kolmogorov-Smirnov comparison of the distributions of reads per bin from an unamplified sample of Halobacterium species NRC-1 with four replicate unamplified control libraries. Bin Size refers to the number of bases comprising each individual bin into which the genome was broken for analysis; 100,000 reads were used for each analysis. As no significant differences were found between the distributions, ranked bias values (derived from D statistics) were assumed equivalent and not assigned. Table S2. Read distributions between unamplified Campylobacter samples. Kolmogorov-Smirnov comparison of the distributions of reads per bin from an unamplified sample of Campylobacter jejuni with four replicate unamplified control libraries. Bin Size refers to the number of bases comprising each individual bin into which the genome was broken for analysis; 100,000 reads were used for each analysis. As no significant differences were found between the distributions, ranked bias values (derived from D statistics) were assumed equivalent and not assigned. [file 1471-2164-7-216-S1.doc]

| Table S1. Kolmogorov-Smirnov comparison of the distributions of reads per bin from an unamplified sample of *Halobacterium* species NRC-1 with four replicate unamplified control libraries. Bin Size refers to the number of bases comprising each individual bin into which the genome was broken for analysis; 100,000 reads were used for each analysis. As no significant differences were found between the distributions, ranked bias values (derived from D statistics) were assumed equivalent and not assigned. | | | | |
| --- | --- | --- | --- | --- |
| **Unamplified Control versus:** | **Replicate 2** | **Replicate 3** | **Replicate 4** | **Replicate 5** |
| **Bin Size (bp)** | 10 | | | |
| **Number of Bins** | 257102 | | | |
| **Number of Reads** | 100000 | | | |
| **D Statistic** | 0.0008 | 0.0006 | 0.0003 | 0.0007 |
| **P value** | 0.999 | 0.999 | 0.999 | 0.999 |
| **Ranked Bias (5 is lowest)** | NA | NA | NA | NA |
|  |  |  |  |  |
|  |  |  |  |  |
| **Bin Size (bp)** | 100 | | | |
| **Number of Bins** | 25711 | | | |
| **Number of Reads** | 100000 | | | |
| **D Statistic** | 0.005 | 0.002 | 0.003 | 0.004 |
| **P value** | 0.926 | 0.999 | 0.999 | 0.995 |
| **Ranked Bias (5 is lowest)** | NA | NA | NA | NA |
|  |  |  |  |  |
|  |  |  |  |  |
| **Bin Size (bp)** | 2571 | | | |
| **Number of Bins** | 1001 | | | |
| **Number of Reads** | 100000 | | | |
| **D Statistic** | 0.033 | 0.021 | 0.026 | 0.033 |
| **P value** | 0.648 | 0.980 | 0.888 | 0.648 |
| **Ranked Bias (5 is lowest)** | NA | NA | NA | NA |
|  |  |  |  |  |
|  |  |  |  |  |
| **Bin Size (bp)** | 25711 | | | |
| **Number of Bins** | 100 | | | |
| **Number of Reads** | 100000 | | | |
| **D Statistic** | 0.1 | 0.11 | 0.07 | 0.1 |
| **P value** | 0.967 | 0.699 | 0.581 | 0.967 |
| **Ranked Bias (5 is lowest)** | NA | NA | NA | NA |

| Table S2. Kolmogorov-Smirnov comparison of the distributions of reads per bin from an unamplified sample of *Campylobacter jejuni* with four replicate unamplified control libraries. Bin Size refers to the number of bases comprising each individual bin into which the genome was broken for analysis; 100,000 reads were used for each analysis. As no significant differences were found between the distributions, ranked bias values (derived from D statistics) were assumed equivalent and not assigned. | | | | |
| --- | --- | --- | --- | --- |
| **Unamplified Control versus:** | **Replicate 2** | **Replicate 3** | **Replicate 4** | **Replicate 5** |
| **Bin Size (bp)** | 10 | | | |
| **Number of Bins** | 164149 | | | |
| **Number of Reads** | 100000 | | | |
| **D Statistic** | 0.0005 | 0.0006 | 0.0007 | 0.0008 |
| **P value** | 1 | 1 | 0.999 | 0.999 |
| **Ranked Bias (5 is lowest)** | NA | NA | NA | NA |
|  |  |  |  |  |
|  |  |  |  |  |
| **Bin Size (bp)** | 100 | | | |
| **Number of Bins** | 16415 | | | |
| **Number of Reads** | 100000 | | | |
| **D Statistic** | 0.005 | 0.002 | 0.004 | 0.003 |
| **P value** | 0.987 | 0.999 | 0.999 | 0.999 |
| **Ranked Bias (5 is lowest)** | NA | NA | NA | NA |
|  |  |  |  |  |
|  |  |  |  |  |
| **Bin Size (bp)** | 1641 | | | |
| **Number of Bins** | 1001 | | | |
| **Number of Reads** | 100000 | | | |
| **D Statistic** | 0.026 | 0.027 | 0.033 | 0.027 |
| **P value** | 0.888 | 0.860 | 0.648 | 0.859 |
| **Ranked Bias (5 is lowest)** | NA | NA | NA | NA |
|  |  |  |  |  |
|  |  |  |  |  |
| **Bin Size (bp)** | 16413 | | | |
| **Number of Bins** | 100 | | | |
| **Number of Reads** | 100000 | | | |
| **D Statistic** | 0.059 | 0.079 | 0.069 | 0.089 |
| **P value** | 0.994 | 0.909 | 0.968 | 0.817 |
| **Ranked Bias (5 is lowest)** | NA | NA | NA | NA |
